# Knockdown of ATRX enhances radiosensitivity in glioblastoma

**DOI:** 10.1186/s41016-024-00371-6

**Published:** 2024-06-19

**Authors:** Yue Zhao, Yifei Chen, Ruoyu Liu, Minghang Liu, Na You, Kai Zhao, Jiashu Zhang, Bainan Xu

**Affiliations:** 1 Medical School of Chinese PLA, Beijing, 100853 China; 2 Department of Emergency Medicine, Hainan Hospital of Chinese PLA General Hospital, Sanya, 572014 Hainan China; 3 Department of Neurosurgery, The First Medical Center, Chinese PLA General Hospital, Beijing, 100853 China; 4 Center of Cognition and Brain Science, Beijing Institute of Basic Medical Sciences, Beijing, 100850 China

**Keywords:** Glioblastoma, ATRX, Radiosensitivity, Knockdown

## Abstract

**Background:**

Glioblastoma are highly malignant type of primary brain tumors. Treatment for glioblastoma multiforme (GBM) generally involves surgery combined with chemotherapy and radiotherapy. However, the development of tumoral chemo- and radioresistance induces complexities in clinical practice. Multiple signaling pathways are known to be involved in radiation-induced cell survival. However, the role of alpha-thalassemia X-linked mutant retardation syndrome (ATRX), a chromatin remodeling protein, in GBM radioresistance remains unclear.

**Methods:**

In the present study, the ATRX mutation rate in patients with glioma was obtained from The Cancer Genome Atlas, while its expression analyzed using bioinformatics. Datasets were also obtained from the Gene Expression Omnibus, and ATRX expression levels following irradiation of GBM were determined. The effects of ATRX on radiosensitivity were investigated using a knockdown assays.

**Results:**

The present study demonstrated that the ATRX mutation rate in patients with GBM was significantly lower than that in patients with low-grade glioma, and that patients harboring an ATRX mutation exhibited a prolonged survival, compared with to those harboring the wild-type gene. Single-cell RNA sequencing demonstrated that ATRX counts increased 2 days after irradiation, with ATRX expression levels also increasing in U-251MG radioresistant cells. Moreover, the results of in vitro irradiation assays revealed that ATRX expression was increased in U-251MG cells, while ATRX knockdown was associated with increased levels of radiosensitivity.

**Conclusions:**

High ATRX expression levels in primary GBM may contribute to high levels of radioresistance. Thus ATRX is a potential target for overcoming the radioresistance in GBM.

**Supplementary Information:**

The online version contains supplementary material available at 10.1186/s41016-024-00371-6.

## Background

Glioblastoma multiforme (GBM) accounts for 48.6% of all malignant central nervous system tumors. Despite multiple treatment options, including surgical resection, radiation therapy, and chemotherapy, the prognosis of GBM remains poor [1–3]. Ionizing radiation eradicates GBM cells by through inducing DNA damage while simultaneously triggering multiple radioresistance signaling pathways, contributing to the limited efficacy of radiotherapy used in GBM treatment [4–10]. Targeting of these signaling pathways could potentially in enhance the radiosensitivity of GBM cells [4, 7, 11, 12].

The mutation status of the Alpha-thalassemia X-linked mutant retardation syndrome (ATRX) was incorporated into the glioma diagnostic algorithm in 2016 [13]. Notably, GBM with the wild-type ATRX was found to be more common in adults with primary GBM. The ATRX mutations occurs at high frequencies in both low-grade gliomas (71%) and secondary GBM (57%) [14]. Isocitrate dehydrogenase (IDH) mutant GBM with ATRX loss exhibits a higher survival rate than the IDH mutant with the wild-type ATRX gene [15]. ATRX is a chromatin remodeling protein that plays an important role in the deposition of the histone variant H3.3 [16]. It has been implicated in various pathways involved in the DNA damage response (DDR), including the replication stress response [17–19], homologous recombination (HR) [20, 21], and nonhomologous end joining (NHEJ) [22]. Genomic profiling of ATRX-deficient adult high-grade gliomas revealed the genetic characteristics of homologous recombination repair [23]. ATRX plays a critical role in double-stranded break (DSB) repair [22]. However, its role in oncogenesis remains unknown [24]. Targeting ATRX and its pathways may improve the radiosensitivity of glioblastoma.

Previous studies have primarily focused on gliomas with ATRX inactivating mutations that leading to resistance to chemotherapeutic agents [25, 26]. ATRX deficiency impairs the process of NHEJ and enhances the sensitivity to DNA-damaging agents [22]. Loss of ATRX also confers sensitivity to poly(ADP)-ribose polymerase inhibitors, which is linked to an increase in replication stress [26]. However, the role of ATRX in the radioresistance of wild-type GBM remains unclear.

The present study aimed to investigate the mutation rate and expression level of ATRX in GBM using bioinformatics analysis. In addition, ATRX expression was determined in GBM cell lines after flowing ionizing irradiation. Cell transfection was performed to investigate the effect of ATRX knockdown on the radiosensitivity of GBM cells.

## Methods

### Bioinformatics analysis of ATRX mutation and expression in GBM

The Cancer Genome Atlas (TCGA) database was searched using the following criteria: project, “TCGA-low-grade glioma (LGG)” or “TCGA-GBM”; data.category, “Simple Nucleotide Variation”; data.type, “Masked Somatic Mutation”; and access, “open.” The R packages maftools (version 2.14.0, R Foundation for Statistical Computing, Vienna, Austria) was subsequently used to analyze the gene mutation status in LGG and GBM. while the Kaplan-Meier and log-rank methods were used to analyze cumulative incidence between ATRX wild-type and mutant genes. Mutation types were further isolated to focus on single-nucleotide polymorphism (SNP) and deletions (DEL), for a more comprehensive survival analysis.

Finally, ATRX expression levels were determined in two datasets (GSE162931 and GSE206917) extracted from the Gene Expression Omnibus (GEO). Gene expression profiling analysis was performed on primary GBM tissue samples and the corresponding paired recurrent tissue samples in the GSE206917 dataset [27]. Moreover, single-cell RNA sequencing profiling was performed on 150,000 single cells obtained from three patient-derived GBM cells in the GSE162931 dataset [28].

### Cell culture and irradiation exposure models

GBM cell lines (U-251MG and LN229) were obtained from the American Type Culture Collection, and these were authenticated by STR profiling (Figs. S1 and S2). All cells were cultured in Dulbecco’s modified Eagle’s medium (DMEM; Gibco; Invitrogen; Thermo Fisher Scientific, Inc.) supplemented with 10% fetal bovine serum (FBS; Gibco; Invitrogen; Thermo Fisher Scientific, Inc.) and 1% penicillin–streptomycin solution (Sigma-Aldrich; Merck KGaA). Cells were cultured in an incubator at 37 ℃ with 5% CO_2_. The U-251MG and LN229 cells were exposed to a single dose of 10-Gy radiation, as described in a previous report [29], and subsequently cultured at 37 ℃ with 5% CO_2_. All cell lines in the radiation group were irradiated at the Beijing Institute of Radiation Medicine (Beijing, China). Blank cell lines that did not received no radiation were used as controls. All experiments were performed in triplicate.

### Reverse transcription-quantitative (RT-q)PCR

Total RNA was extracted from the GBM cells using 200-μl TRIzol^®^ reagent (Sigma-Aldrich: Merck KGaA). Total RNA was reverse transcribed using Superscript III Reverse Transcriptase (Invitrogen; Thermo Fisher Scientific, Inc.) and random primers. qPCR was subsequently performed using a reaction system comprising 500-ng cDNA, 250-nM upstream and downstream primers, and 12.5 μl of 2× SYBR Green real-time PCR Master Mix (TOYOBO Life Science). The levels of ATRX mRNA were normalized to those of GAPDH. Relative mRNA levels (ATRX/GAPDH) were defined as the ratio of normalized ATRX mRNA levels in the experimental and control groups. All primers used in the present study were designed and synthesized by Tsingke Biological Technology (Table 1).

**Table 1:** Primers used in RT-qPCR

Gene	Primer	Sequence (5′-3′)
GAPDH	Forward	ATGGGGAAGGTGAAGGTCG
GAPDH	Reverse	GGGGTCATTGATGGCAACAATA
ATRX	Forward	ACGGCGTTAGTGGTTTGTCCTC
ATRX	Reverse	GCAGCATGTAGCTTCTCTCCTG

*RT-qPCR* reverse transcription-quantitative PCR, *ATRX* alpha-thalassemia X-linked mutant retardation syndrome

### Small interfering (si)RNA transfection

An siRNA mix was created by combining 6 μl of siRNA (100 μM; Table 2) with 500 μl of Opti-MEM solution (Gibco; Invitrogen; Thermo Fisher Scientific, Inc.) An oligofectamine mix was simultaneously created by mixing 10 μl of Lipofectamine^®^ 2000 (Invitrogen; Thermo Fisher Scientific, Inc.) with 500 μl of Opti-MEM. Both the mixtures were incubated at room temperature for 5 min. The two mixtures were then combined and incubated at room temperature 20 min. For transfection, the cells were cultured until 50–75% confluency was reached. The cells were then washed three times with DMEM without FBS, and DMEM (without FBS) was added directly into the culture plate. Notably, each 100-mm culture plate contained 500 μl of the final mix, comprising siRNA, Lipofectamine, and Opti-MEM. The cells were then incubated at 37 °C for 6 h. Subsequently, 10 ml of DMEM with 20% FBS was added to each culture plate, and the cells were incubated at 37 °C for further 24 h prior to the subsequent experiments. All siRNA and negative controls (NCs) used in this the present study were designed and synthesized by Suzhou GenePharma Co., Ltd.

**Table 2:** siRNAs used in the present study

siRNA	siRNA sequence (5′-3′)
ATRX-siRNA1-sense	CGAAAGGAGUUGUCCACAATT
ATRX-siRNA1-antisense	UUGUGGACAACUCCUUUCGTT
ATRX-siRNA2-sense	CCAAAGAAGACUAGUUCAATT
ATRX-siRNA2-antisense	UUGAACUAGUCUUCUUUGGTT
ATRX-siRNA3-sense	GGCUCAUCUUGCAUUGGAATT
ATRX-siRNA3-antisense	UUCCAAUGCAAGAUGAGCCTT
ATRX-siRNA4-sense	CGACUUGCAAUGAAUCAAATT
ATRX-siRNA4-antisense	UUUGAUUCAUUGCAAGUCGTT

*siRNA*, small interfering RNA; *ATRX*, alpha-thalassemia X-linked mutant retardation syndrome

### Colony formation assay

The colony formation assay was performed in 6-well plates. Cell suspensions were diluted to obtain a seeding concentration of 0.2 × 10^4^/ml for U-251MG cells and 0.5 × 10^4^/ml for LN229 cells. Following seeding, all cells were incubated for 15 days. After incubation, the cell medium was removed, and cells were washed with PBS. In total, 2-ml 0.5% crystal violet was added to all cell suspensions, after which the plates were incubated at room temperature for 30 min. The cells were then washed with tap water to remove the crystal violet solution. The plates were dried at room temperature, and the number of colonies was determined. A colony was defined as a group containing of at least 50 aggregates.

### Statistical analysis

All data are expressed as the mean ± standard error of the mean (SEM). Statistical analyses were conducted using GraphPad Prism software (version, 8.0; GraphPad Software, Inc.). Two-tailed unpaired Student’s *t*-tests were used to analyze differences between two groups, and one-way or two-way ANOVA were used to analyze the differences between multiple groups with post hoc comparisons using the Student–Newman–Keuls test. Bioinformatics analyses were performed using R software (version, 4.2.2; R Foundation for Statistical Computing, Vienna, Austria). Kaplan-Meier analysis and log-rank tests were performed using SPSS (version, 26; IBM Corp.). Statistical significance was set at *P* < 0.05.

## Results

### Lower rates of ATRX mutations in primary GBM are associated with reduced survival times

**Fig. 1:**
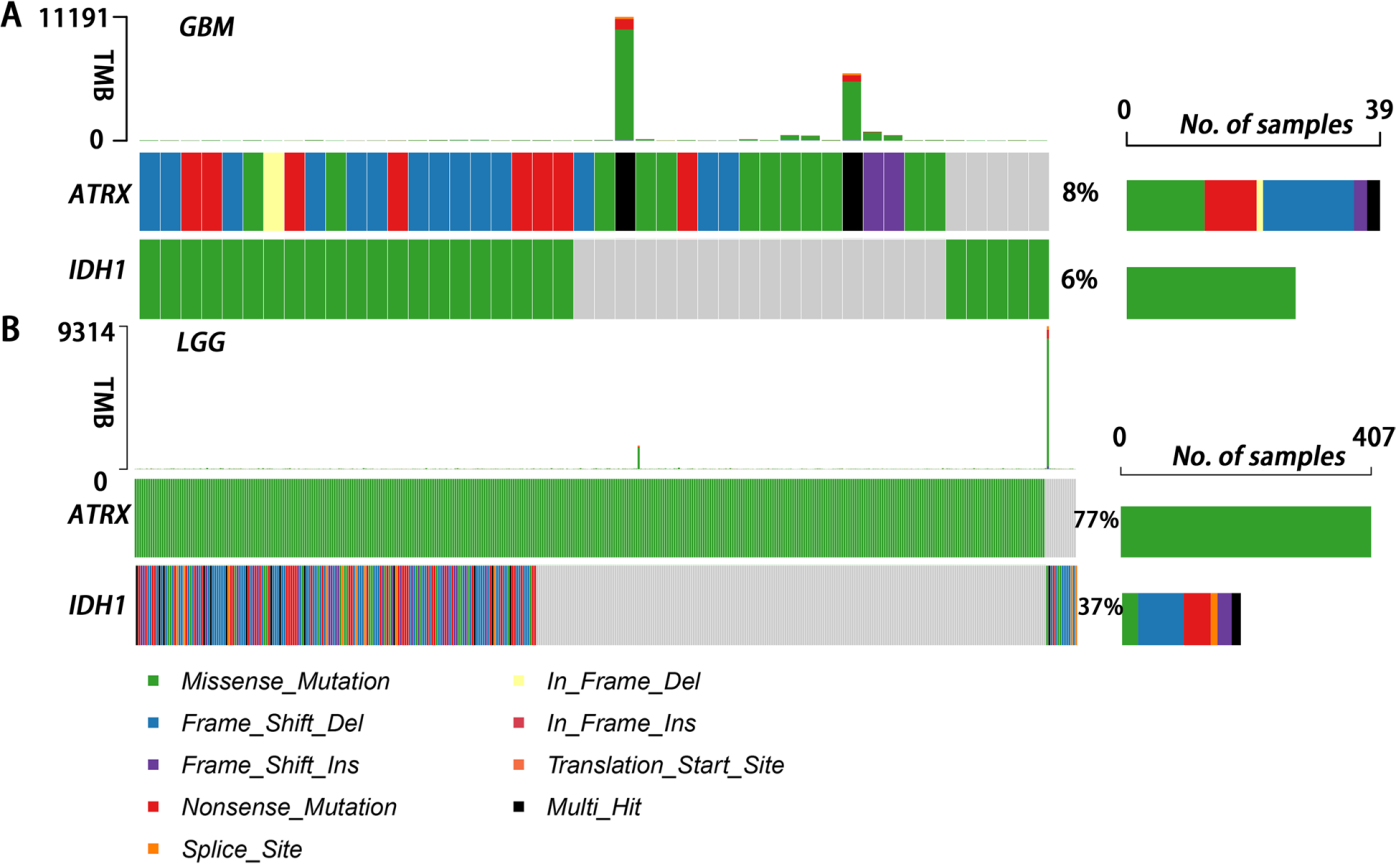
ATRX mutation in **A** GBM and **B** LGG. ATRX mutation rate in patients with GBM (8%) was significantly lower than in patients with LGG (77%). Missense mutations, frameshift deletions, and in-frame insertions were the major mutations observed in ATRX in patients with GBM. ATRX mutations observed in patients with LGG were all missense mutations. ATRX, alpha-thalassemia X-linked mutant retardation syndrome; GBM, glioblastoma multiforme; LGG, low-grade glioma

The ATRX mutation status in patients with GBM or LGG in the TCGA database was analyzed using bioinformatics analysis. While the IDH mutation status of tumors further analyzed for comparison. In the TCGA-LGG group, 10,675 gene mutations were detected in 524 patients, while in the TCGA-GBM group, there were 12,803 mutations were identified in 461 patients. Overall, the ATRX mutation rate in patients with GBM was significantly lower(8%), than that in patients with LGG (77% Fig. 1). Further, ATRX and IDH1 mutations do not always occur simultaneously. The results of the present study demonstrated that missense mutations, frameshift deletions, and in-frame insertions were the major ATRX mutations observed in ATRX in patients with GBM (Fig. 1A). In contrast, ATRX mutations observed in patients with LGG were all missense mutations.

**Fig. 2:**
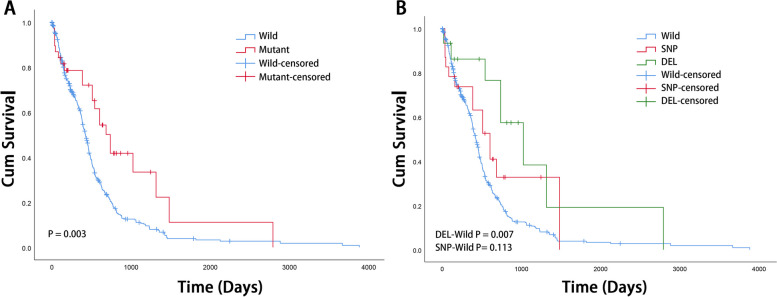
Kaplan-Meier analysis and log-rank test of overall survival between ATRX mutation and wild-type group. **A** Patients with an ATRX mutation exhibited a significantly prolonged survival time compared with patients with the ATRX wild-type gene (*P* = 0.003). **B** Patients with an ATRX deletion exhibited a significantly prolonged survival time compared with patients with the ATRX wild-type gene (*P* = 0.007). No significant differences were observed in patients with ATRX SNPs compared with patients with the ATRX wild-type gene (*P* = 0.113). ATRX, alpha-thalassemia X-linked mutant retardation syndrome; SNP, single-nucleotide polymorphism

Subsequently, survival analysis was performed to determine whether the ATRX mutation status was associated with the overall survival of patients with GBM. The results of the Kaplan-Meier analysis revealed that patients with GBM harboring an ATRX mutation exhibited a significantly prolonged survival time (log-rank test, *P* = 0.003), compared to those with wild-type ATRX (Fig. 2A). The subgroups of SNPs and DELs in the observed mutations was further investigated. Compared to patients with the wild-type ATRX gene, patients with an ATRX deletion exhibited a significantly prolonged survival time (log-rank test, *P* = 0.007). However, no significant differences were observed between patients with an ATRX SNP and those with the wild-type ATRX (log-rank test, *P* = 0.113).

### ATRX expression levels are increased in GBM models and recurrent GBM tissue samples

**Fig. 3:**
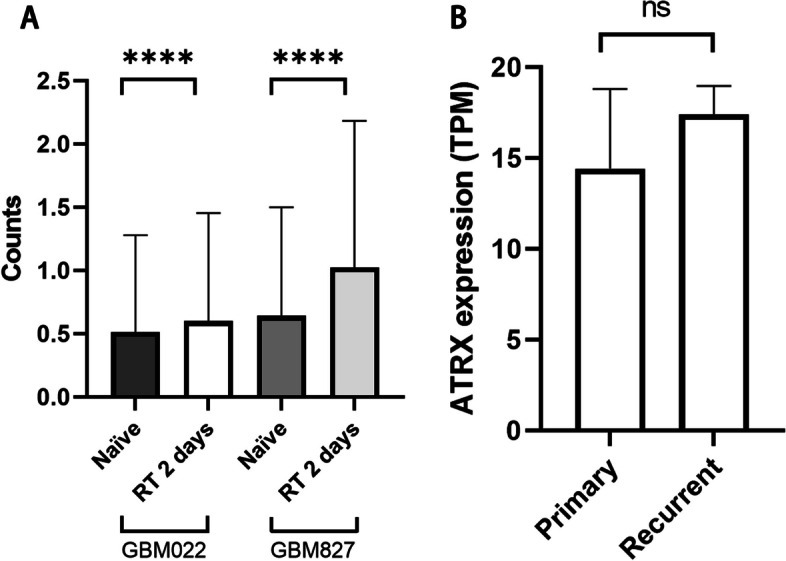
ATRX expression levels analyzed in GSE162931 and GSE206917 datasets using bioinformatics. **A** Single-cell RNA sequencing profiling in GSE162931 datasets revealed that ATRX expression levels were increased 2 days after radiation. **B** Bulk RNA sequencing analysis revealed that ATRX expression levels were increased in recurrent GBM tissue samples; however, the difference was not statistically significant. ****P < 0.0001. ATRX, alpha-thalassemia X-linked mutant retardation syndrome; GBM, glioblastoma multiforme

Single-cell RNA sequencing profiling was performed using the GSE162931 datasets obtained from the GEO database. Orthotopic patient-derived GBM models (827 and 022 GBM cells) were irradiated with 10 Gy irradiation and harvested after two days. The results of the single-cell RNA sequencing demonstrated that ATRX counts were significantly increased 2 days after irradiation (Fig. 3A, *P*< 0.001). Bulk RNA-seq sequencing analysis using the GSE206917 dataset further revealed that ATRX expression levels were increased in recurrent GBM tissue samples; however, the difference was not statistically significant (Fig. 3B, *P*> 0.05).

### ATRX expression levels in U-251 MG cells are increased following irradiation

**Fig. 4:**
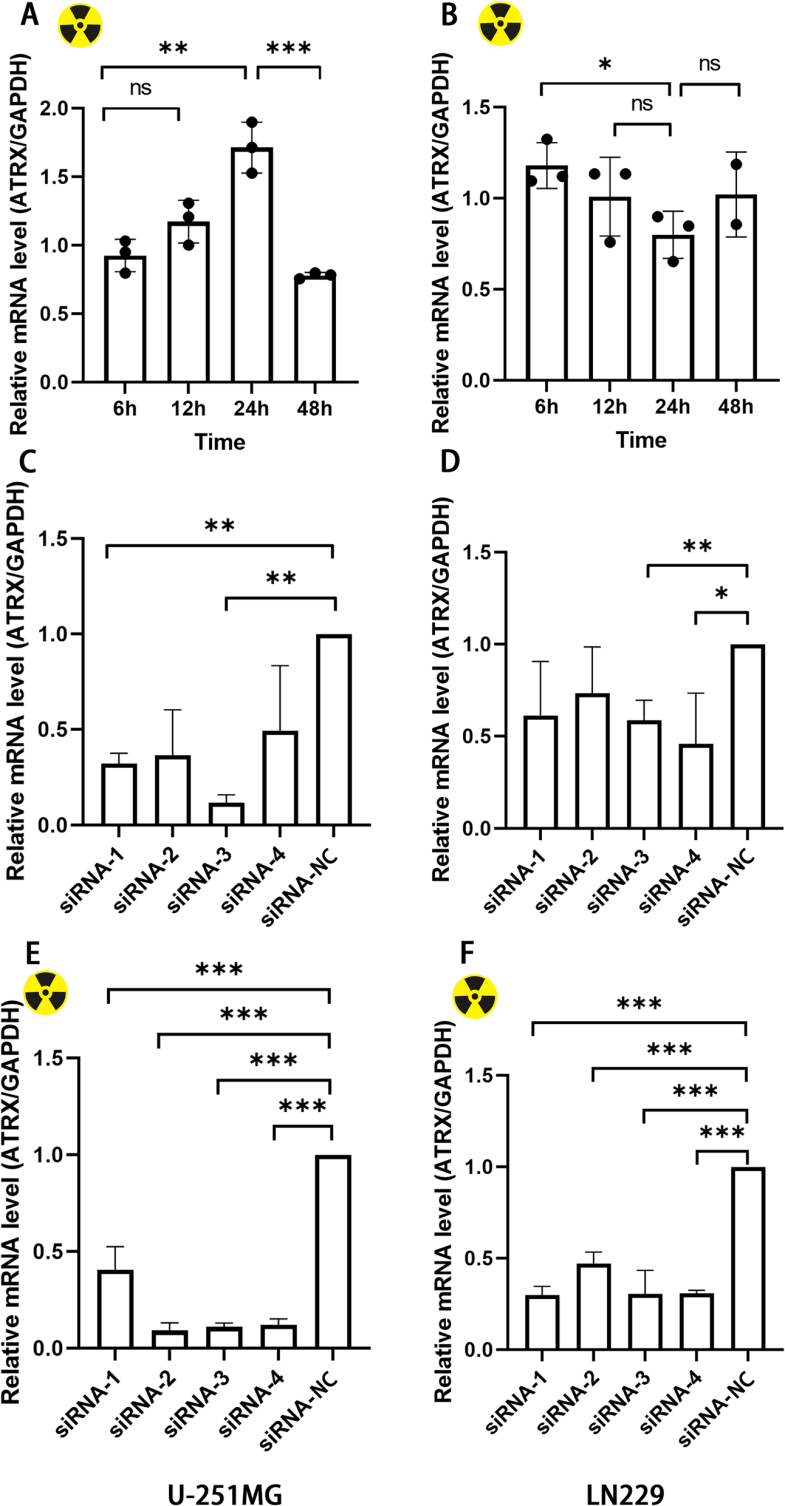
ATRX expression levels measured by reverse transcription-quantitative PCR after irradiation and siRNA transfection assays. **A** and **B** ATRX expression levels were significantly increased following irradiation in U-251MG cells, and the expression levels peaked at 24 h after radiation. Relative ATRX/GAPDH mRNA expression levels were defined as the ratio of normalized ATRX mRNA levels in the experimental group to the control group. **C** and **D** Transfection efficiency of siRNA 1–3 reached 90–60% in U-251MG cells. Transfection efficiency of siRNA 1–3 reached 28–59% in LN229 cells. **E** and **F** ATRX expression levels were not increased in all four siRNA groups in U-251MG and LN229 cells following irradiation. *P < 0.05, **P < 0.01, ***P < 0.001. siRNA, small interfering RNA; ATRX, alpha-thalassemia X-linked mutant retardation syndrome

To determine ATRX expression levels in GBM cells following irradiation, U-251MG and LN229 GBM cells were exposed to a single dose of *γ*-radiation. while, ATRX mRNA expression levels were measured at 6h, 12h, 24h, and 48h after irradiation. The results of the present study demonstrated that ATRX expression levels in U-251MG cells were significantly increased following irradiation. Notably, ATRX expression reached the highest level at 24h post-irradiation, after which it gradually decreased (Fig. 4A). This observed increase was not evident in LN229 cells (Fig. 4B), suggesting that does radiation did not induce increased ATRX expression in LN229 cells.

### ATRX knockdown inhibits the radiation-induced upregulation of ATRX expression

This, study subsequently aimed to determine whether ATRX knockdown affects the radiosensitivity of GMB cells. In the present study, Four siRNAs were used to knockdown ATRX in U-251MG and LN229 cells, the transfection efficiency is shown in Fig. 4C and D. In U-251MG cells, stable ATRX knockdown was achieved following transfection with siRNA 1–3, with a transfection efficiency was 68–90%. In LN229 cells, the transfection efficiency was 28%–59%. At 24h following ATRX knockdown, GBM cells were irradiated, and ATRX expression levels were subsequently measured using RT-qPCR. In U-251MG and LN229 cells, ATRX expression was not altered following irradiation with of the four siRNA groups (Fig. 4E and 4F).

### ATRX knockdown increases the radiosensitivity of U-251MG cells

**Fig. 5:**
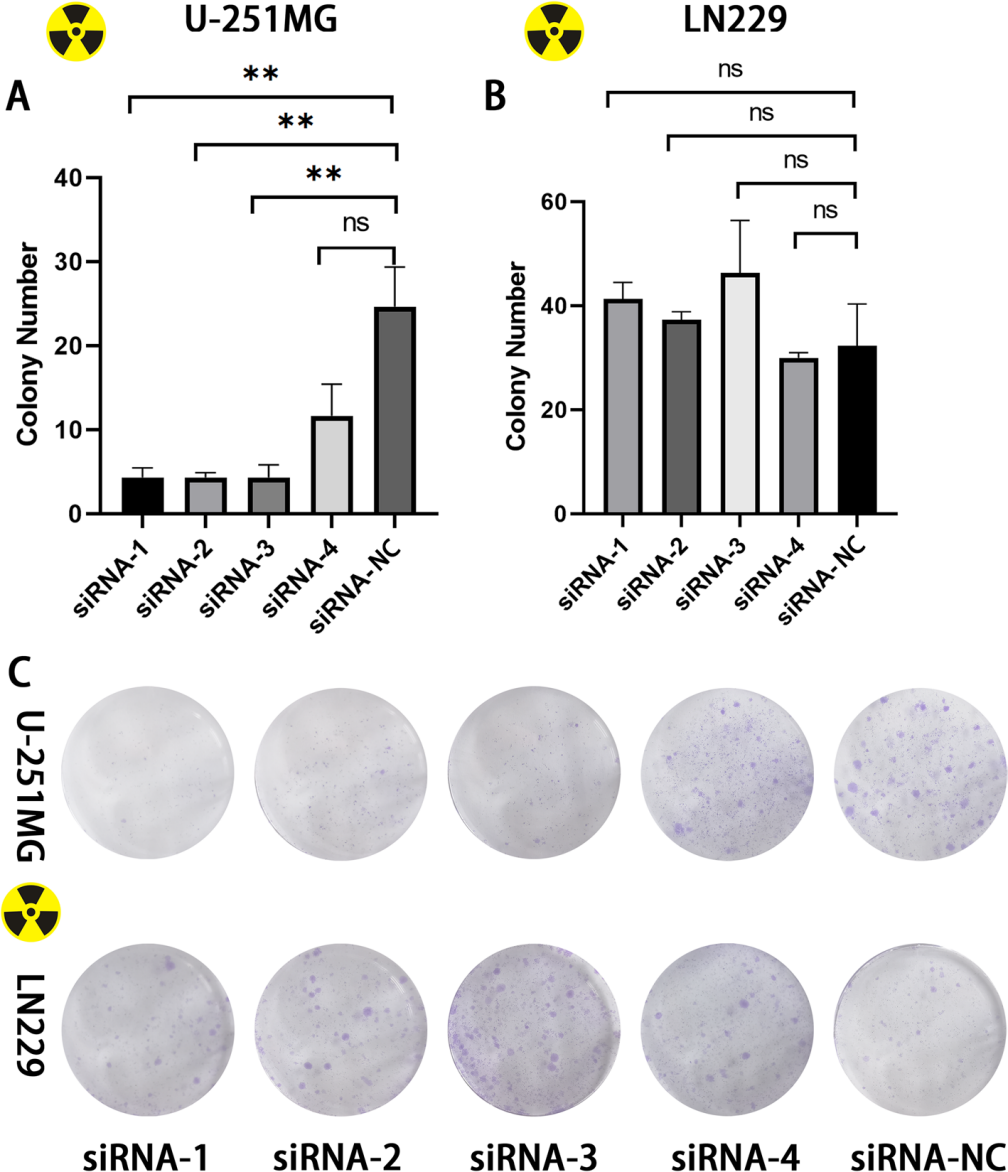
ATRX knockdown increases the radiosensitivity of U-251MG cells. Colony formation assays were carried out using U-251MG and LN229 cells. Transfection with siRNA 1–3 significantly inhibited U-251MG cell proliferation compared with siRNA-NC. But cell proliferation of LN229 was not inhibited. **P < 0.01. ATRX, alpha-thalassemia X-linked mutant retardation syndrome; siRNA, small interfering RNA; NC, negative control

The present study aimed to determine whether ATRX knockdown increased the radiosensitivity of GBM cells. Results of the colony formation assay demonstrated that ATRX knockdown using siRNA 1–3 significantly inhibited the proliferation of U-251MG cells, indicating that ATRX knockdown may increase the radiosensitivity of U-251MG cells (Fig. 5). In the siRNA 4 group, there was no significant difference in the colony number of U-251MG cells compared to the NC group, possibly due to the low transfection efficiency of U-251MG cells. Moreover the proliferation of LN229 cell was not inhibited.

## Discussion

As a chromatin remodeling protein, ATRX plays an important role in double-stranded break repair in glioma [16, 22]. ATRX is directly recruited directly to sites of DNA damage, and plays a noncanonical function role in guarding genomic stability [17]. ATRX expression lever vary across different grades of gliomas. Notably, decreased ATRX mRNA expression has been found to be indicative of low-grade astrocytoma [30]. ATRX mutations are more common in secondary GBMs than in primary tumors [31]. One previous study demonstrated that patients in the ATRX-low group exhibited significantly prolonged overall survival compared to those in the ATRX-high group [32].

Through bioinformatics analysis of LGG and GBM cases obtained from TCGA datasets, the present study revealed that the ATRX mutation rate in patients with GBM was significantly lower than that in patients with LGG. Moreover, we found that ATRX and IDH1 mutations do not always occur simultaneously in GBM tumors. The results of a previous study investigating 163 adult patients with GBM demonstrated that ATRX-/IDH1+ only accounted for 4.3% of the cases, whereas ATRX−/IDH1− accounted for 11% [33]. The results of the Kaplan-Meier analysis in the present study revealed that patients harboring an ATRX mutation exhibited a significantly prolonged survival time compared to those harboring with the wild-type gene, which was comparable with the results those of a previous study [33]. In addition, another previous study revealed that pediatric patients with HGG harboring an ATRX mutation exhibited a prolonged survival time [34]. Similarly, our results also revealed that patients with an ATRX deletion exhibited a significantly prolonged survival time compared to those with the ATRX wild-type gene.

GBM cells expressing with wild-type ATRX gene exhibit increased resistance to irradiation; however, the specific underlying molecular mechanism remains unclear. An improved response to irradiation has been observed in mice harboring ATRX-deficient tumors [22]. The results of the present study indicate that ATRX expression in U-251MG cells was significantly increased following irradiation, while the expression levels peaked at 24h following irradiation. The results of the colony formation assay revealed that siRNA-mediated knockdown of ATRX enhanced radiosensitivity. Notably, ATRX expression did not increased following irradiation of LN229 cells, while radiosensitivity was not enhanced following ATRX knockdown. As such, we hypothesized that increased expression of ATRX induced by radiation in GBM indicates of radioresistance. Further investigation into the specific molecular mechanisms and downstream signaling pathways in U-251MG and LN229 cells are required.

Previous studies have described the biological role of ATRX [16, 35, 36]. In one such study, Voon et al. [37] demonstrated that ATRX knockout results in the depletion of H3.3 and the loss of the H3K9me3 heterochromatin modifications at the methylated allele of imprinted differentially methylated regions [37]. ATRX further binds to zinc finger proteins (ZNFs) to maintain genomic stability by presering H3K9me3 [38]. The localization of chromobox homolog 5 (CBX5) in telomeres is dependent on the association of ATRX with histone H3.3 [37, 39, 40]. ATRX-RNA interacts to regulates PRC2 localization to a subset of polycomb target genes [41]. ATRX further promotes chromatin reconstitution during the DNA repair synthesis step of homologous recombination [20]. Multiple pro-survival signaling pathways, including those mediated by ATM, ATR, AKT, ERK, YAP, and NF-κB, which promote the activation of DNA damage checkpoint or DNA repair, induction of autophagy, and/or inhibition of apoptosis. These pathways contribute to the intrinsic radioresistance of cancer cells [4]. In another study Qin et al found that ATRX-deficient GBM cells exhibited enhanced sensitivity to irradiation. Notably, ATRX-deficiency causes exhibit dysregulation of cell cycle phase transitions is mediated by checkpoint kinase 1 [29], which may be a potential a target for overcoming radioresistance in GBM harboring the wild-type ATRX gene.

ATRX knockdown induces telomere dysfunction, and this phenotype significantly decreases the enrichment inCBX5 at the telomeres [40]. ATRX knockdown has further been shown to result in a perturbed S-phase progression and increased sensitivity to replication stress [17]. Cells lacking ATRX are more sensitive to DNA-damaging agents [42]. ATRX deficiency hinders the NHEJ process and increases sensitivity to DNA-damaging agents that induce double-stranded DNA breaks in mouse tumors [22]. ATRX deficiency specifically amplifies DNA damage and cellular apoptosis both *in vitro* and *in vivo* [43]. In multiple GBM models, ATRX loss has been associated with dysregulation of the cell cycle phase transition in response to irradiation [29]. Targeting ATRX and its downstream pathways may thus have exhibit potential in overcoming the radioresistance of GBM harboring the ATRX-wild gene.

The present study has several limitations. Firstly, although targeting ATRX may reduce radioresistance in GBM, the degree to which ATRX contributes to radioresistance varies depending on the GBM type. Thus, further investigations with multiple glioblastoma cell lines and orthotopic tumor formation experiments in mice are required. Secondy, the siRNA-mediated knockdown of a target gene induces transcript degradation. In addition, siRNA-mediated knockdown is transient, and GBM cells may regain ATRX expression. Future studies should involve permanent ATRX knockout assays using CRISPR-Cas9 to obtain more high-fidelity results. Moreover, our study showed that the proliferation of U-251MG cells following ATRX knockdown was found to be decreased after radiation exposure. However, it should be notede that a previous study implicated ATRX in the apoptotic process, showing that siRNA-mediated knockdown of ATRX leads to an increase in apoptotic cells [32]. Loss of ATRX has also been shown to augment the ability of glioma cells to induce T-cell apoptosis [44]. Thus, further investigations are required to determine the impact of ATRX knockdown on apoptosis and necrosis in GBM cells.

## Conclusions

The present study revealed that the ATRX mutation rate in patients with GBM was significantly lower than that in patients with LGG, while and patients harboring an ATRX mutation exhibited a significantly prolonged survival time, compared to those harboring the ATRX wild-type gene. ATRX expression levels were also significantly increased in U-251MG cells following irradiation, while siRNA-mediated ATRX knockdown enhanced the radiosensitivity of GBM cells. Thus, the high expression levels of ATRX induced by radiation in GBM may be indicative of radioresistance.

### Supplementary Information

Supplementary Material 1.

Supplementary Material 2.

## Data Availability

The datasets used and/or analyzed during the current study are available from the corresponding author on reasonable request.

## Abbreviations

HGG: High-grade glioma
LGG: Low-grade glioma
ATRX: Alpha-thalassemia X-linked mutant retardation syndrome
GBM: Glioblastoma multiforme
