# Supplementary figures and images for: Knockdown of ATRX enhances radiosensitivity in glioblastoma
Source: Chin Neurosurg J. 2024 Jun 19;10:19. doi: 10.1186/s41016-024-00371-6 (PMC11186225; doi:10.1186/s41016-024-00371-6)

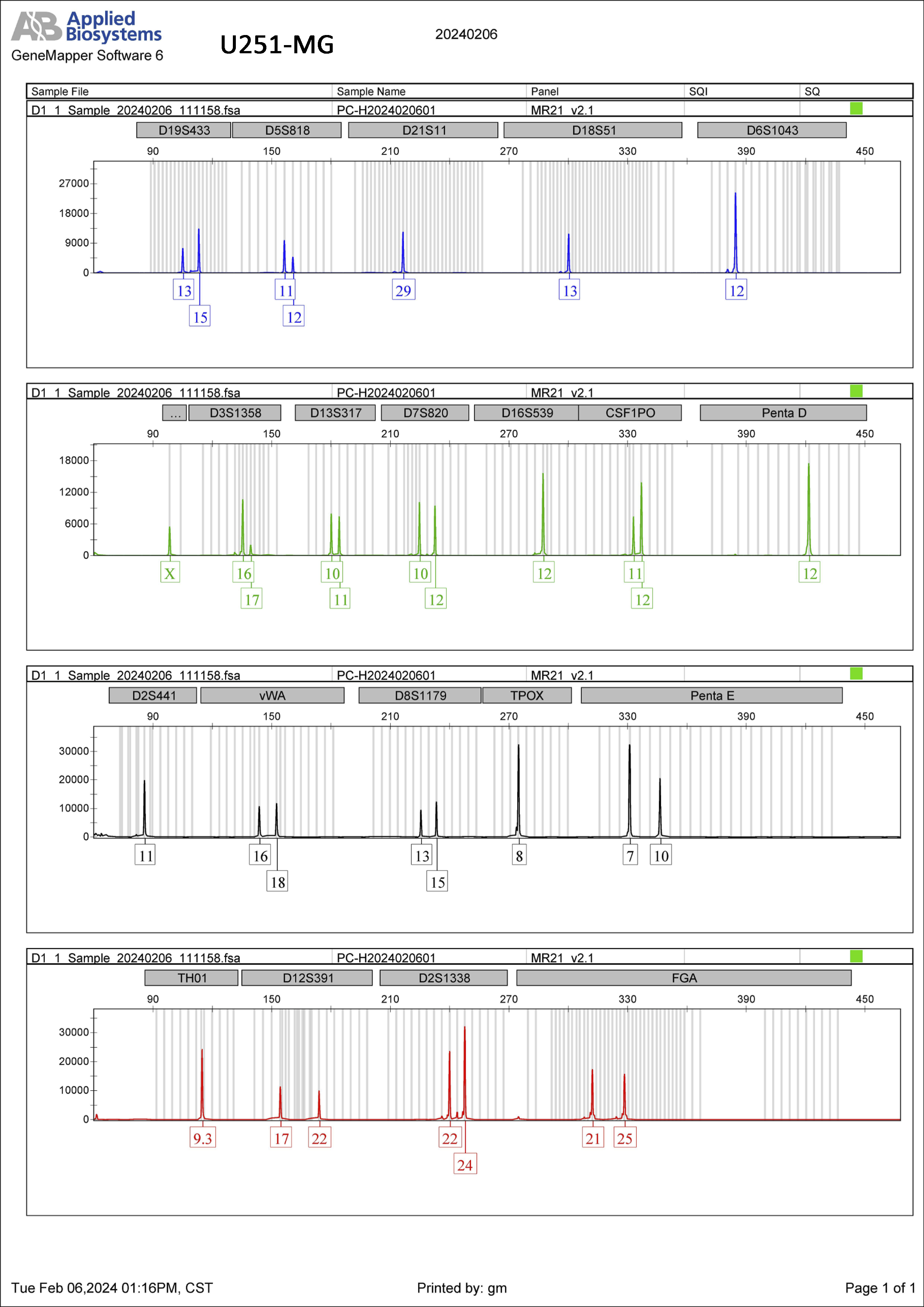

Supplement: Supplementary file 1 — Supplementary Material 1. [file 41016_2024_371_MOESM1_ESM.tif]

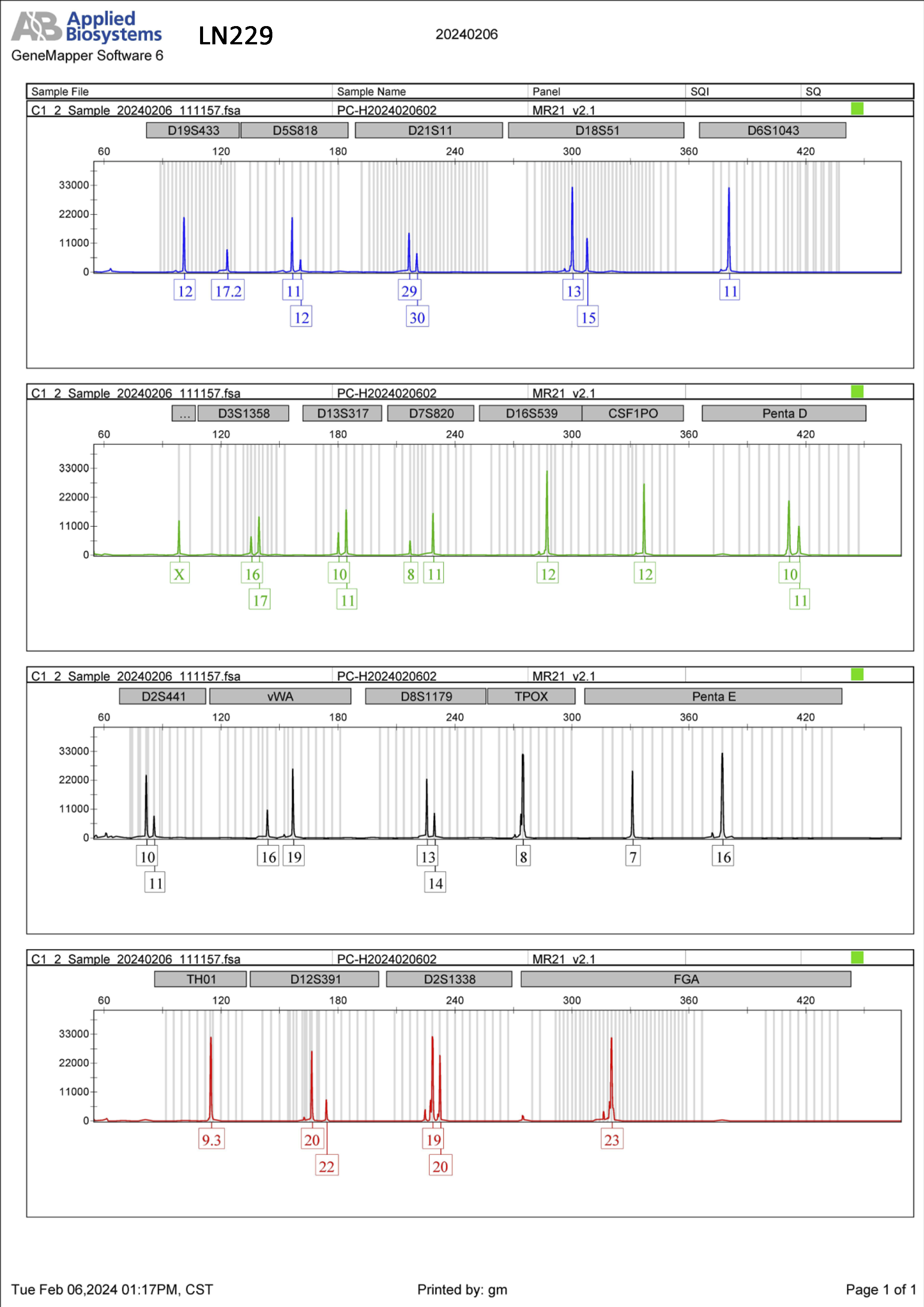

Supplement: Supplementary file 2 — Supplementary Material 2. [file 41016_2024_371_MOESM2_ESM.tif]
